# Time-segmented, feedback-guided tiered warming during radical esophagectomy: a single-center retrospective cohort study

**DOI:** 10.3389/fonc.2026.1920051

**Published:** 2026-09-30

**Authors:** Yanshu Liu, Jianlan Ren, Lu Xiao, Juan Wang, Ling Wang, Yu Yi, Jie Cheng, Min Hu

**Affiliations:** 1 Department of Operating Room, The affiliated Hospital of Southwest Medical University, Luzhou, Sichuan, China; 2 Department of Anesthesiology, The affiliated Hospital of Southwest Medical University, Luzhou, Sichuan, China; 3 Department of Neurology, The affiliated Hospital of Southwest Medical University, Luzhou, Sichuan, China

**Keywords:** esophageal cancer, esophagectomy, inadvertent hypothermia, perioperative quality improvement, perioperative warming, public health

## Abstract

**Objective:**

To evaluate whether a protocolized intraoperative temperature-management strategy based on time-segmented warning thresholds and tiered, feedback-guided warming interventions reduces hypothermia and improves early postoperative recovery in patients undergoing radical esophagectomy.

**Methods:**

This single-center retrospective comparative cohort study included 116 adults who underwent elective radical esophagectomy at The affiliated Hospital of Southwest Medical University between August 2024 and January 2026 and were managed with either a time-segmented, feedback-guided tiered warming protocol (n = 58) or conventional warming (n = 58). Core temperature was monitored continuously with a lower esophageal probe until the start of esophageal mobilization and with a nasopharyngeal probe thereafter, and was recorded every 15 min. All warming adjustments were implemented manually by the clinical team according to predefined thresholds. The primary endpoint was intraoperative hypothermia, defined as a core temperature below 36 °C at any time from incision to skin closure. Secondary endpoints included severe hypothermia, nadir temperature, hypothermia burden, postoperative shivering, time to extubation, post-anesthesia care unit (PACU) recovery time, coagulation indices, 30-day complications, length of stay, and hospital cost.

**Results:**

Intraoperative hypothermia occurred in 53 of 58 control patients (91.4%) and 5 of 58 intervention patients (8.6%) (risk ratio 0.09, 95% CI 0.04-0.22; P < 0.001). Severe hypothermia below 35.5 °C developed in 27 control patients (46.6%) and in none of the intervention patients. Median nadir core temperature was 35.55 °C in the control group and 36.27 °C in the intervention group (P < 0.001). Compared with conventional warming, the protocol reduced postoperative shivering (5.2% vs 51.7%), shortened time to extubation by 8.9 min, lowered the rate of any postoperative complication (34.5% vs 62.1%; P = 0.005), shortened hospital stay (12.2 vs 13.5 days; P = 0.010), and reduced mean in-hospital cost by 8,911 CNY (P < 0.001). The treatment effect was materially unchanged in adjusted analyses using Firth penalized logistic regression (adjusted odds ratio 0.004, 95% CI 0.000-0.024) and modified Poisson regression (adjusted risk ratio 0.10, 95% CI 0.04-0.23).

**Conclusion:**

In adults undergoing radical esophagectomy, a time-segmented, feedback-guided tiered warming protocol substantially reduced intraoperative hypothermia and was associated with faster early recovery, fewer postoperative complications, shorter hospitalization, and lower cost. Because the intervention was a multicomponent warming bundle, the contributions of its individual components cannot be separated, and the associations with complications, hospital stay, and cost should be regarded as hypothesis-generating. The protocol uses standard perioperative equipment and can be implemented without specialized hardware.

## Introduction

Esophageal cancer remains a major health burden, particularly in East Asia. Recent global estimates indicate more than half a million new cases and more than 440,000 deaths each year, with China accounting for a large proportion of this burden (1, 2). For resectable disease, radical esophagectomy remains a key component of curative treatment despite advances in multimodal therapy (3). The operation is physiologically demanding, often prolonged, and involves both the thoracic and abdominal cavities; even in experienced centers, pulmonary complications and anastomotic failure remain important concerns (4, 5).

This setting is especially prone to inadvertent hypothermia. General anesthesia widens the inter-threshold range for thermoregulation, blunts vasoconstriction, and promotes rapid redistribution of heat from the core to the periphery (6). During long operations, these effects are compounded by large exposed fields, evaporative heat loss, unwarmed irrigation, and fluid administration. National and regional surveys from China have shown that intraoperative hypothermia remains common in routine practice and that active warming is not applied consistently (7, 8). In radical esophagectomy, where operative exposure and duration are both substantial, the risk is likely to be even higher than that reported in mixed general surgical cohorts.

Even mild perioperative hypothermia has clinically relevant consequences. Randomized and observational studies have associated lower perioperative temperature with higher rates of wound infection, greater blood loss and transfusion, delayed emergence, and longer hospitalization (9–14). Esophagectomy-specific studies have reported similar findings, linking lower intraoperative temperature to early postoperative adverse events, including pulmonary complications and other major morbidity (15, 16).

Current guidance therefore favors prevention rather than delayed rescue. Active surface warming, warmed fluids, prewarming, and continuous temperature monitoring are recommended as routine components of perioperative care (17–19). In practice, however, temperature management in many operating rooms remains reactive: warming is started after induction, maintained at a fixed setting, and intensified only after the core temperature has already fallen below 36 °C. The PROTECT trial did not show that aggressive warming reduced major cardiovascular events in a broad non-cardiac surgical population (20), but that result does not resolve how best to manage temperature during prolonged, high-risk oncologic procedures in which sustained heat loss is expected.

Structured, feedback-guided temperature management offers a practical way to address this problem. Fully automated closed-loop systems have improved the consistency of anesthetic titration and other perioperative processes (21), and experimental work suggests that automated thermal control can be implemented with standard warming hardware (22). Such automated systems, however, are not yet available for routine thermal care. Our center therefore developed a pragmatic, protocolized warming strategy for radical esophagectomy that combines time-segmented warning thresholds with tiered warming interventions implemented manually by the clinical team, matched to the expected pattern of heat loss during surgery. In this retrospective cohort study, we compared patients managed with this strategy against contemporaneous patients managed with conventional warming, hypothesizing that the protocol would be associated with less intraoperative hypothermia and better short-term postoperative outcomes.

## Materials and methods

### Study design and participants

We conducted a single-center retrospective comparative cohort study of patients who underwent elective radical esophagectomy at The Affiliated Hospital of Southwest Medical University between August 2024 and January 2026. Eligible patients were 18 to 80 years of age, had biopsy-confirmed esophageal squamous cell carcinoma or adenocarcinoma, and underwent elective radical esophagectomy with curative intent. Patients were excluded if they underwent emergency surgery, had a preoperative core temperature outside 35.5-37.5 °C, had a documented history of malignant hyperthermia or thyrotoxicosis, had severe hepatic or renal dysfunction (Child-Pugh C or estimated glomerular filtration rate < 30 mL/min/1.73 m²), had cardiopulmonary disease severe enough to preclude standard general anesthesia, or were pregnant. The study was designed and reported with reference to the STROBE statement and the Declaration of Helsinki (23, 24). The study protocol was approved by the institutional ethics committee of The Affiliated Hospital of Southwest Medical University (ethics approval No. KY2026396), and the approval covered the retrospective use of routinely collected, de-identified clinical data. As a retrospective observational study without prospective assignment of an intervention, it did not meet the ICMJE definition of a clinical trial, and prospective trial registration was therefore not applicable; no public trial registration was obtained. Written informed consent for surgery and for the use of de-identified clinical data was obtained from all patients.

### Cohort formation and outcome ascertainment

During the study period, both the time-segmented, feedback-guided tiered warming protocol and conventional warming were in contemporaneous routine use at our center, and the warming strategy each patient received was determined by the managing clinical team’s practice rather than by study procedures. Intraoperative temperature data were retrieved from the electronic anesthesia record, and postoperative outcomes were ascertained from the electronic medical record and routine follow-up documentation.

### Intervention and control protocols

Core temperature was monitored continuously with a lower esophageal temperature probe placed after tracheal intubation, with the tip positioned in the distal esophagus and displayed continuously during the initial operative stages. Because the esophagus is mobilized and transected during the procedure, the esophageal probe was withdrawn at the start of esophageal mobilization, and core temperature was monitored with a nasopharyngeal probe advanced to the posterior nasopharynx for the remainder of the operation; the two sites provide clinically interchangeable estimates of core temperature, and the same monitoring sequence was applied in both groups. Readings from the active probe were recorded at 15-min intervals. In the intervention group, management followed a protocolized, feedback-guided strategy with phase-specific alert thresholds and tiered warming responses. Temperature acquisition, threshold assessment, and adjustment of warming devices were performed manually by the circulating nurse and the attending anesthesiologist according to the written protocol; no component of this loop was automated. During Phase I (0–30 min from incision), the warning threshold was 36.5 °C and the alarm threshold was 36.2 °C. During Phase II (30–60 min), the thresholds were 36.3 °C and 36.0 °C. During Phase III (60–180 min), the thresholds were 36.2 °C and 36.0 °C. During Phase IV (>180 min), the warning threshold remained 36.2 °C and the alarm threshold was 35.9 °C. All intervention patients received background warming from skin preparation onward, including at least 15 min of prewarming, a forced-air blanket set at 38 °C, a fluid warmer at 39 °C, and an ambient room temperature of at least 22 °C. Crossing the warning threshold triggered escalation of the forced-air unit to 43 °C and the fluid warmer to 41 °C. Crossing the alarm threshold prompted the addition of an under-body resistive mattress and warming of irrigation fluids. If temperature did not recover within 15 min, the room temperature was increased by 1 °C, draping was reinforced, and warm-air insufflation was considered when technically feasible. De-escalation occurred once the core temperature exceeded 36.3 °C on two consecutive measurements. Control patients received the institutional standard of care: passive insulation with drapes and blankets, forced-air warming at a fixed setting of 38 °C, intravenous fluid warming at 37 °C, and discretionary escalation only after the temperature had fallen below 36.0 °C. All warming adjustments in both groups were implemented manually; the protocol did not involve automated device control.

### Anesthesia and perioperative management

Anesthesia was standardized as total intravenous anesthesia with propofol target-controlled infusion (target plasma concentration 2.5-4.0 μg/mL), remifentanil (0.1-0.3 μg/kg/min), and cisatracurium for neuromuscular blockade. Thoracic epidural analgesia (T6-T7) was used at the discretion of the attending anesthesiologist. Standard monitoring included electrocardiography, invasive arterial pressure, pulse oximetry, capnography, bispectral index monitoring, and continuous core temperature monitoring using the esophageal-to-nasopharyngeal probe sequence described above.

### Surgical teams and postoperative management

All operations were performed by one of three attending thoracic surgical teams at our center, each with an annual volume of more than 50 esophagectomies, using standardized institutional techniques. Reconstruction used a gastric conduit in all patients, with an intrathoracic circular-stapled esophagogastric anastomosis for Ivor-Lewis procedures and a cervical anastomosis for McKeown and transhiatal procedures; lymphadenectomy followed a standardized two-field template unless clinically indicated otherwise. The distribution of surgical approaches was balanced between groups (**Table 1**). Postoperative care in both groups followed a standardized, enhanced-recovery-based institutional pathway, including protocolized analgesia, early mobilization, chest physiotherapy, and stepwise nutritional management. Enteral nutrition was initiated on postoperative day 1–2 via a nasoenteral feeding tube, oral fluids were permitted after clinical and radiographic assessment of the anastomosis at approximately postoperative day 5-7, and diet was advanced in a graded manner. Per institutional routine, patients were contacted by nursing staff at days 7 and 14 after discharge and reviewed in the outpatient clinic at day 30; complications and adherence to postoperative recommendations documented at these contacts were ascertained from the medical records, and 30-day data were complete for all included patients.

**Table 1:** Baseline demographic, clinical, and operative characteristics.

Variable	Control (n = 58)	Intervention (n = 58)	P value
Age (years)	63.6 ± 8.0	58.7 ± 7.8	0.001
Male sex, n (%)	46 (79.3%)	44 (75.9%)	0.824
BMI (kg/m²)	23.4 ± 2.9	22.4 ± 2.4	0.051
ASA class III, n (%)	27 (46.6%)	21 (36.2%)	0.346
Tumor location, n (%)			
Upper third	15 (25.9%)	11 (19.0%)	
Middle third	27 (46.6%)	29 (50.0%)	
Lower third	16 (27.6%)	18 (31.0%)	
Clinical TNM stage, n (%)			
Stage I	11 (19.0%)	15 (25.9%)	
Stage II	26 (44.8%)	22 (37.9%)	
Stage III	21 (36.2%)	21 (36.2%)	
Surgical approach, n (%)			
McKeown	31 (53.4%)	33 (56.9%)	
Ivor-Lewis	24 (41.4%)	24 (41.4%)	
Transhiatal	3 (5.2%)	1 (1.7%)	
Hypertension, n (%)	25 (43.1%)	19 (32.8%)	0.339
Diabetes mellitus, n (%)	14 (24.1%)	9 (15.5%)	0.352
Smoking history, n (%)	29 (50.0%)	27 (46.6%)	0.853
Preoperative hemoglobin (g/L)	130.0 ± 13.7	124.5 ± 12.6	0.028
Preoperative albumin (g/L)	38.2 ± 4.4	38.0 ± 4.3	0.809
General plus epidural anesthesia, n (%)	42 (72.4%)	33 (56.9%)	0.120
Operating-room temperature (°C)	21.9 ± 0.6	22.2 ± 0.6	0.039
Operative duration (min)	277.4 ± 48.7	269.7 ± 38.8	0.347
Baseline core temperature (°C)	36.7 ± 0.2	36.7 ± 0.2	0.674
Intraoperative fluid volume (mL)	2213.6 ± 392.9	2186.6 ± 370.5	0.703

Data are mean ± SD or n (%). BMI, body mass index; ASA, American Society of Anesthesiologists; TNM, tumor-node-metastasis.

### Outcomes

The primary endpoint was intraoperative hypothermia, defined as a core temperature below 36.0 °C at any time between surgical incision and skin closure. Secondary endpoints included severe hypothermia (<35.5 °C), nadir core temperature, mean intraoperative temperature, hypothermia burden (defined as the integrated area below 36.0 °C and expressed as °C·min), time to first hypothermic event, postoperative shivering during the first hour in the PACU, estimated blood loss, allogeneic transfusion, time to tracheal extubation, PACU recovery time, postoperative day 1 prothrombin time (PT) and activated partial thromboplastin time (APTT), 30-day complications (surgical site infection, pulmonary complication, anastomotic leak, cardiac arrhythmia, and any complication), intensive care unit (ICU) stay, total hospital stay, and in-hospital cost.

### Sample size and statistical analysis

An audit of 30 consecutive radical esophagectomies performed at our center before the study period showed an intraoperative hypothermia rate of 72%. For the primary endpoint, 15 patients per group would provide 80% power to detect a reduction to 20% with a two-sided α of 0.05. To improve precision for the secondary outcomes and to allow the planned subgroup analyses, a larger analytic sample of 58 eligible patients per group was included in the analysis. Continuous variables are reported as mean ± SD or median (interquartile range) and were compared with Student’s t test or the Mann-Whitney U test as appropriate. Categorical variables are presented as counts (percentages) and were compared with the chi-square test or Fisher’s exact test. The analysis plan, defined before the analysis was undertaken, specified an adjusted analysis of the primary endpoint including age, sex, BMI, ASA class, operative duration, operating-room temperature, and baseline core temperature as covariates; no covariate was added or removed after the results had been seen. Because the primary endpoint was very common in the control group and rare in the protocol group, conventional maximum-likelihood logistic regression was expected to be vulnerable to sparse-data bias, so the adjusted model was fitted with Firth penalized logistic regression with profile-penalized-likelihood confidence intervals and likelihood-ratio P values. To aid interpretation of a common outcome, we additionally estimated the adjusted risk ratio with modified Poisson regression (log link, robust variance) and the adjusted risk difference with a linear-probability model with robust standard errors. A *post hoc* sensitivity analysis added preoperative hemoglobin to the Firth model to address the observed baseline imbalance in this variable. Model behavior was examined before interpretation: separation was confirmed from the maximum-likelihood iteration history and the associated standard errors, convergence of the penalized model was verified, and the influence of individual observations on the Firth estimates was assessed by leave-one-out refitting. Baseline core temperature is reported per 0.1 °C increment, because the observed range of this variable within the cohort was narrow and an estimate expressed per 1 °C would represent a substantial extrapolation beyond the observed data. Time to first hypothermic event was analyzed with Kaplan-Meier methods and the log-rank test. Predefined subgroup analyses examined age, sex, BMI, ASA class, surgical approach, and operative duration. All tests were two-sided, and P < 0.05 was considered statistically significant.

## Results

### Study cohort and baseline characteristics

A total of 116 patients were included in the analysis, 58 in the control group and 58 in the protocol (intervention) group. All included patients had complete intraoperative temperature records and complete 30-day outcome data. Baseline characteristics are summarized in **Table 1**. The groups were generally similar with respect to BMI, ASA class, tumor location, clinical stage, surgical approach, operative duration, and intraoperative fluid administration. Three baseline imbalances were noted: patients in the intervention group were younger, had lower preoperative hemoglobin values, and underwent surgery in a slightly warmer operating room. Age and operating-room temperature were already included in the *a priori* adjusted analysis of the primary endpoint; preoperative hemoglobin was not part of the *a priori* covariate set and was examined in a *post hoc* sensitivity analysis.

### Intraoperative temperature control

The temperature trajectories diverged early and remained separated throughout the procedure (**Figure 1**). In the control group, core temperature showed the expected two-phase pattern: an early fall during the first 30–60 min, consistent with redistribution, followed by a slower decline during continued cavity exposure. By the third operative hour, mean temperature in the control group had fallen below 36 °C. In contrast, patients managed with the feedback-guided protocol had a smaller initial decrease and returned toward the normothermic range after approximately 90 min. Thermal outcomes are summarized in **Table 2**. Intraoperative hypothermia occurred in 53 of 58 control patients (91.4%) compared with 5 of 58 intervention patients (8.6%) (P < 0.001). Severe hypothermia below 35.5 °C developed in 27 control patients (46.6%) and in none of the intervention patients. Median nadir temperature was 35.55 °C in the control group and 36.27 °C in the intervention group (**Figure 2**). Hypothermia burden also differed markedly between groups, averaging 56.8 ± 54.7 °C·min in controls and 0.9 ± 5.7 °C·min in the intervention arm (P < 0.001).

**Figure 1:**
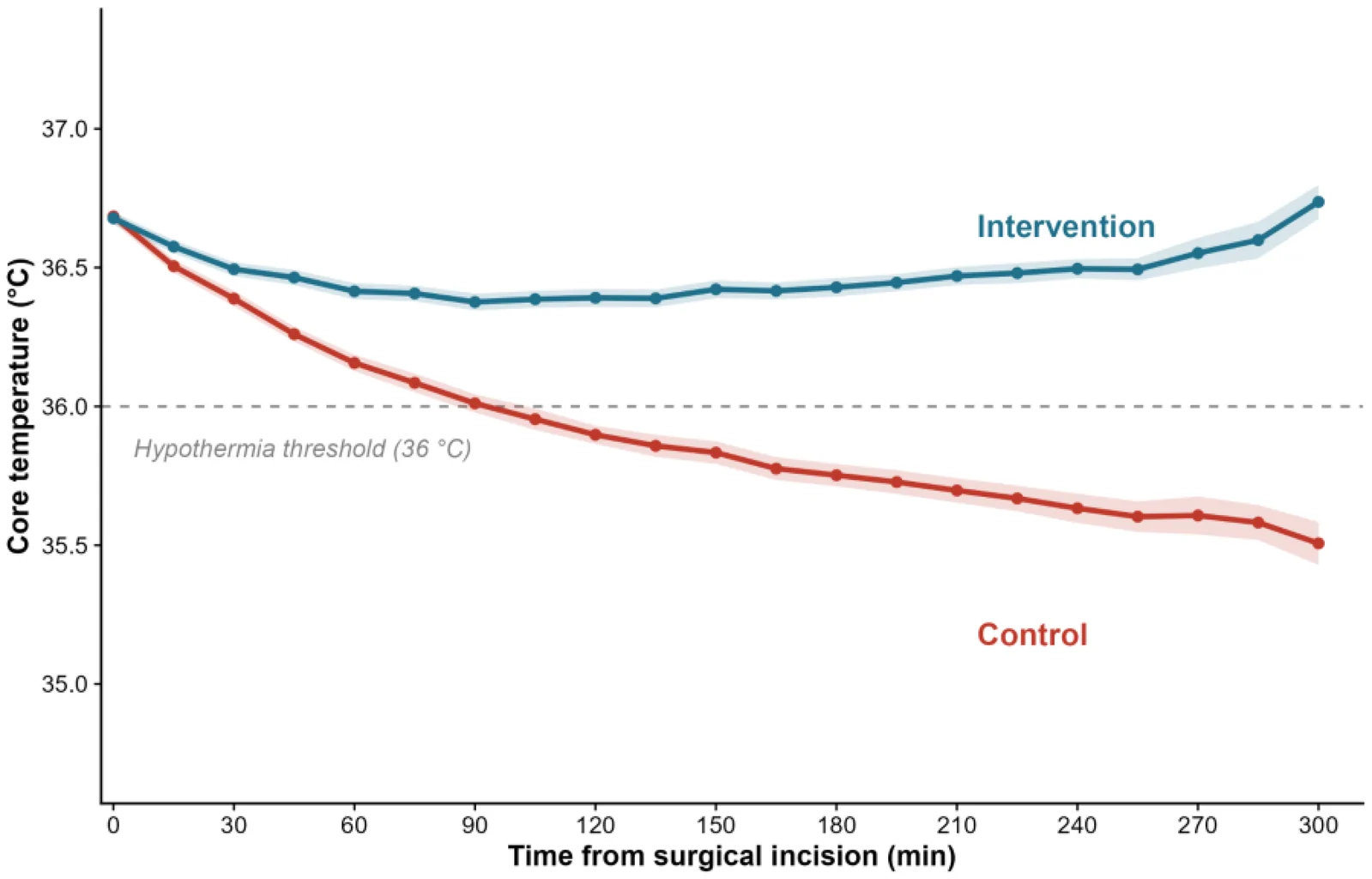
Intraoperative core temperature trajectory in the control and intervention groups. Values are presented as mean ± standard error at 15-min intervals. The dashed line marks the hypothermia threshold of 36 °C.

**Table 2:** Primary and secondary intraoperative and postoperative outcomes.

Variable	Control (n = 58)	Intervention (n = 58)	P value
Primary endpoint			
Intraoperative hypothermia (< 36.0 °C), n (%)	53 (91.4%)	5 (8.6%)	<0.001
Intraoperative thermoregulation			
Severe hypothermia (< 35.5 °C), n (%)	27 (46.6%)	0 (0.0%)	<0.001
Nadir core temperature (°C)	35.5 ± 0.3	36.3 ± 0.2	<0.001
Mean intraoperative core temperature (°C)	36.0 ± 0.3	36.5 ± 0.2	<0.001
Hypothermia burden (°C·min)	56.8 ± 54.7	0.9 ± 5.7	<0.001
Postoperative recovery			
Postoperative shivering, n (%)	30 (51.7%)	3 (5.2%)	<0.001
Time to extubation (min)	29.3 ± 6.1	20.4 ± 7.1	<0.001
PACU recovery time (min)	38.1 ± 7.8	31.4 ± 7.8	<0.001
Bleeding and coagulation			
Estimated intraoperative blood loss (mL)	316.7 ± 84.9	293.3 ± 97.1	0.171
Allogeneic transfusion, n (%)	12 (20.7%)	7 (12.1%)	0.316
Postoperative PT (s)	14.9 ± 1.4	13.6 ± 1.4	<0.001
Postoperative APTT (s)	35.7 ± 3.7	33.0 ± 3.4	<0.001
Length of stay and cost			
ICU stay (days)	2.4 ± 0.8	1.9 ± 0.8	<0.001
Hospital stay (days)	13.5 ± 2.5	12.2 ± 2.4	0.010
Hospital cost (CNY)	90,700.6 ± 10,363.0	81,789.5 ± 9,688.2	<0.001

Data are mean ± SD or n (%). Hypothermia burden was calculated as the integrated area below 36 °C across the operation and is expressed as degree-minutes. PACU, post-anesthesia care unit; PT, prothrombin time; APTT, activated partial thromboplastin time; ICU, intensive care unit; CNY, Chinese yuan.

**Figure 2:**
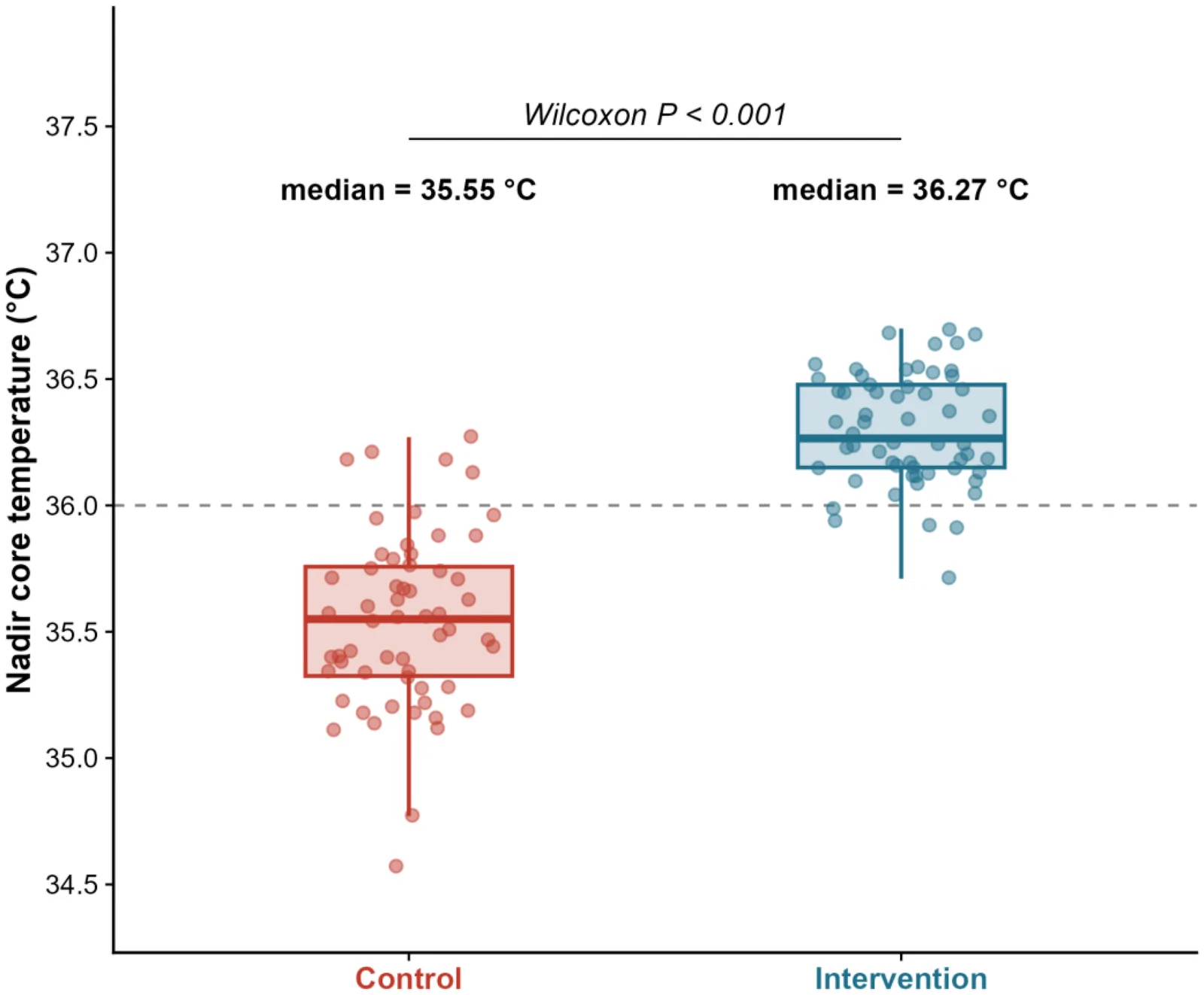
Nadir intraoperative core temperature in each group. Individual points, medians, and the Wilcoxon test result are shown.

### Time to first hypothermic event

Time-to-event analysis showed early and sustained separation between groups (**Figure 3**). At 60 min from incision, about one-third of patients in the control arm had already crossed the 36 °C threshold. By 120 min, cumulative incidence in that group had reached roughly 60%, and by the end of surgery it was 91.4%. In the intervention arm, cumulative incidence remained below 10% throughout the procedure. The log-rank test confirmed a significant difference in time to first hypothermic event (P < 0.001). A complementary cumulative-incidence analysis using fixed denominators showed the same pattern (**Figure 4**). These analyses indicate that the intervention did not simply delay hypothermia, but prevented it in most patients.

**Figure 3:**
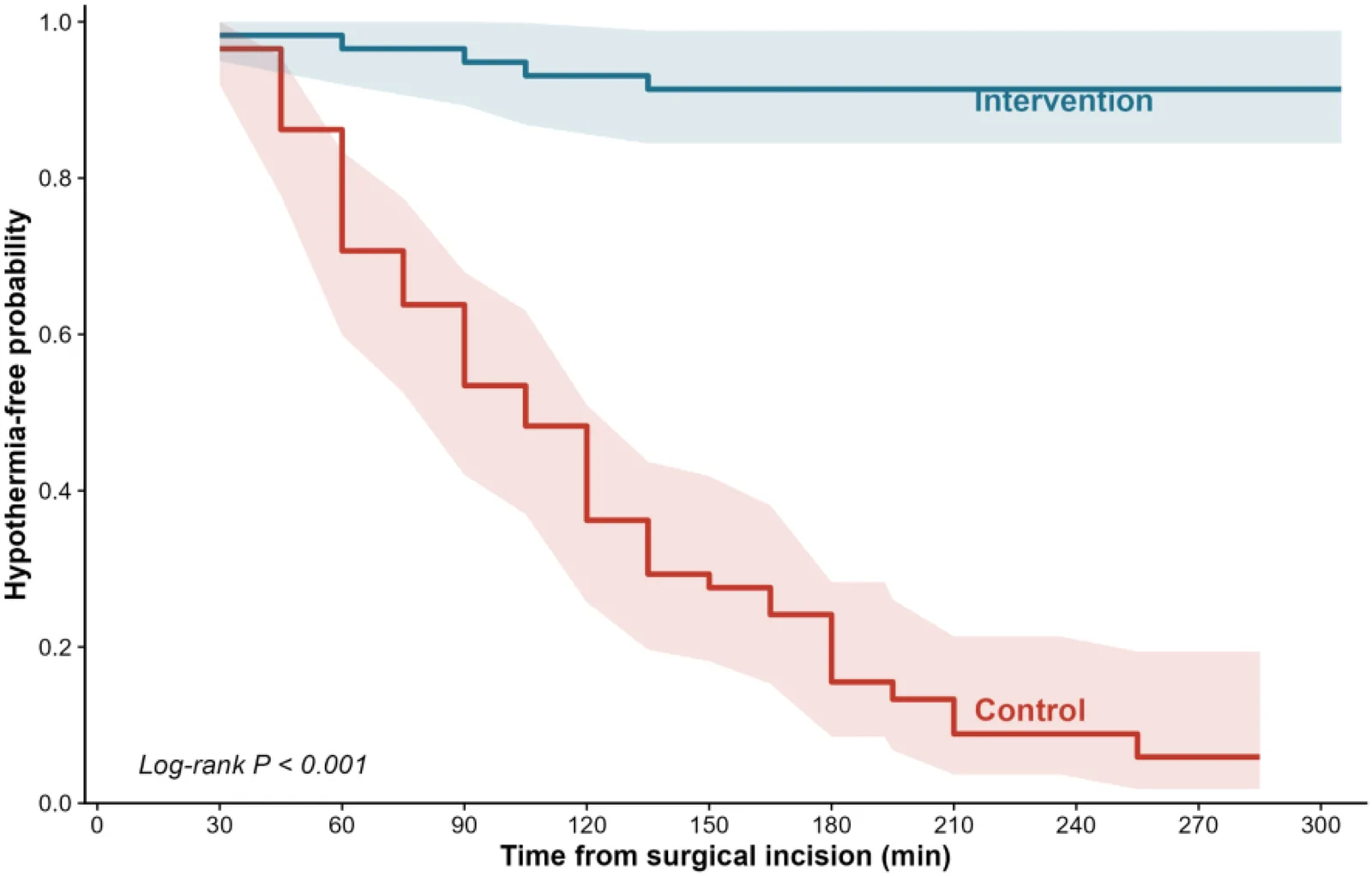
Kaplan-Meier estimate of remaining free from intraoperative hypothermia over time from surgical incision.

**Figure 4:**
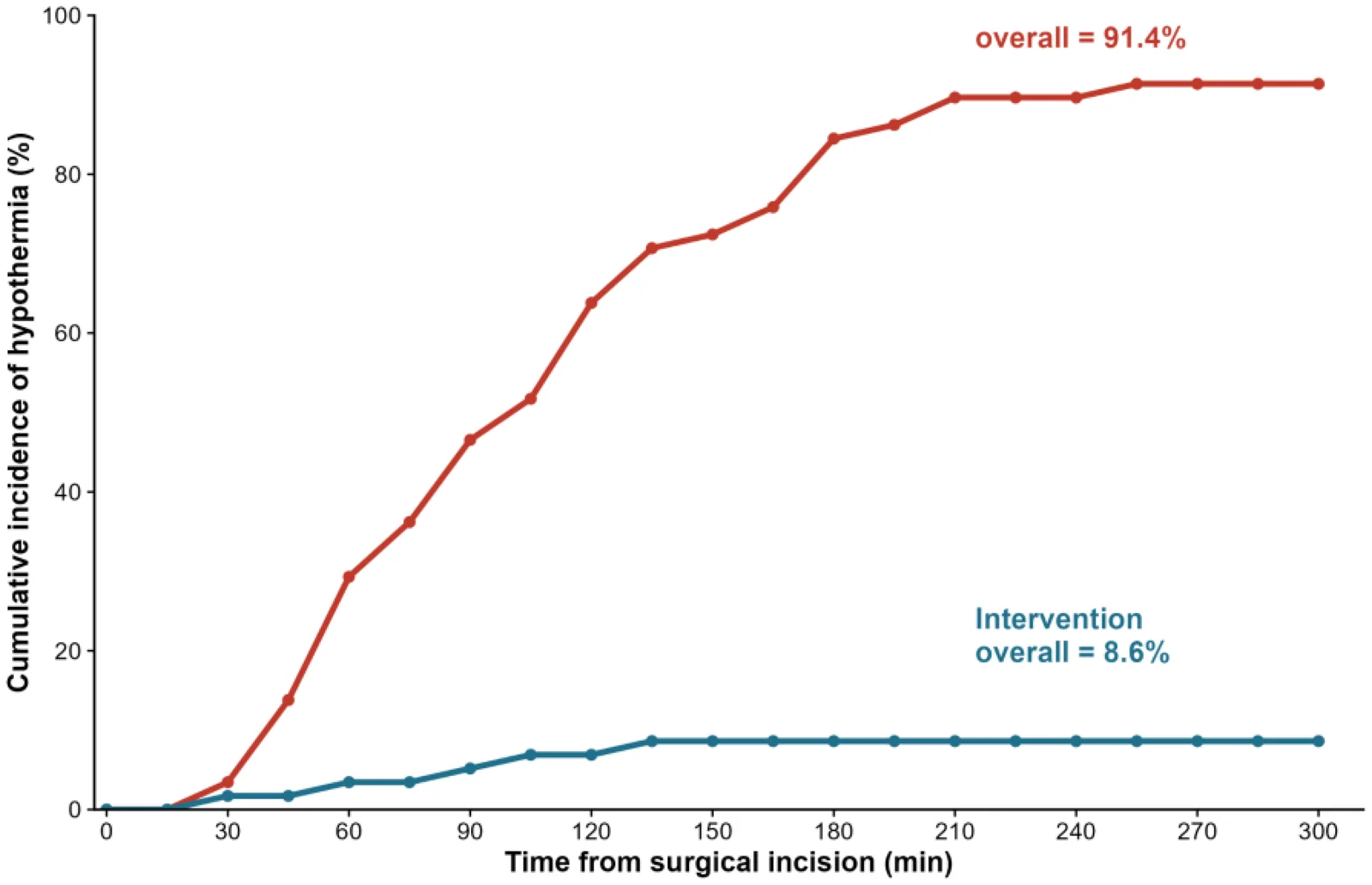
Cumulative incidence of intraoperative hypothermia (< 36 °C) over time with denominators fixed at 58 patients per group.

### Recovery, coagulation, and resource use

The between-group differences extended beyond temperature control itself (**Table 2**; **Figure 5**). Postoperative shivering fell from 51.7% in the control group to 5.2% in the intervention group. Time to extubation was shorter in the intervention group (20.4 ± 7.1 vs 29.3 ± 6.1 min; P < 0.001), as was PACU recovery time (31.4 ± 7.8 vs 38.1 ± 7.8 min; P < 0.001). Postoperative day 1 PT and APTT were statistically longer in the control group, although mean values in both groups remained within or near the reference range, so these differences indicate a measurable thermal effect on coagulation rather than clinically significant hypocoagulation. Estimated blood loss and allogeneic transfusion both trended downward with protocolized warming, although neither difference reached conventional statistical significance.

**Figure 5:**
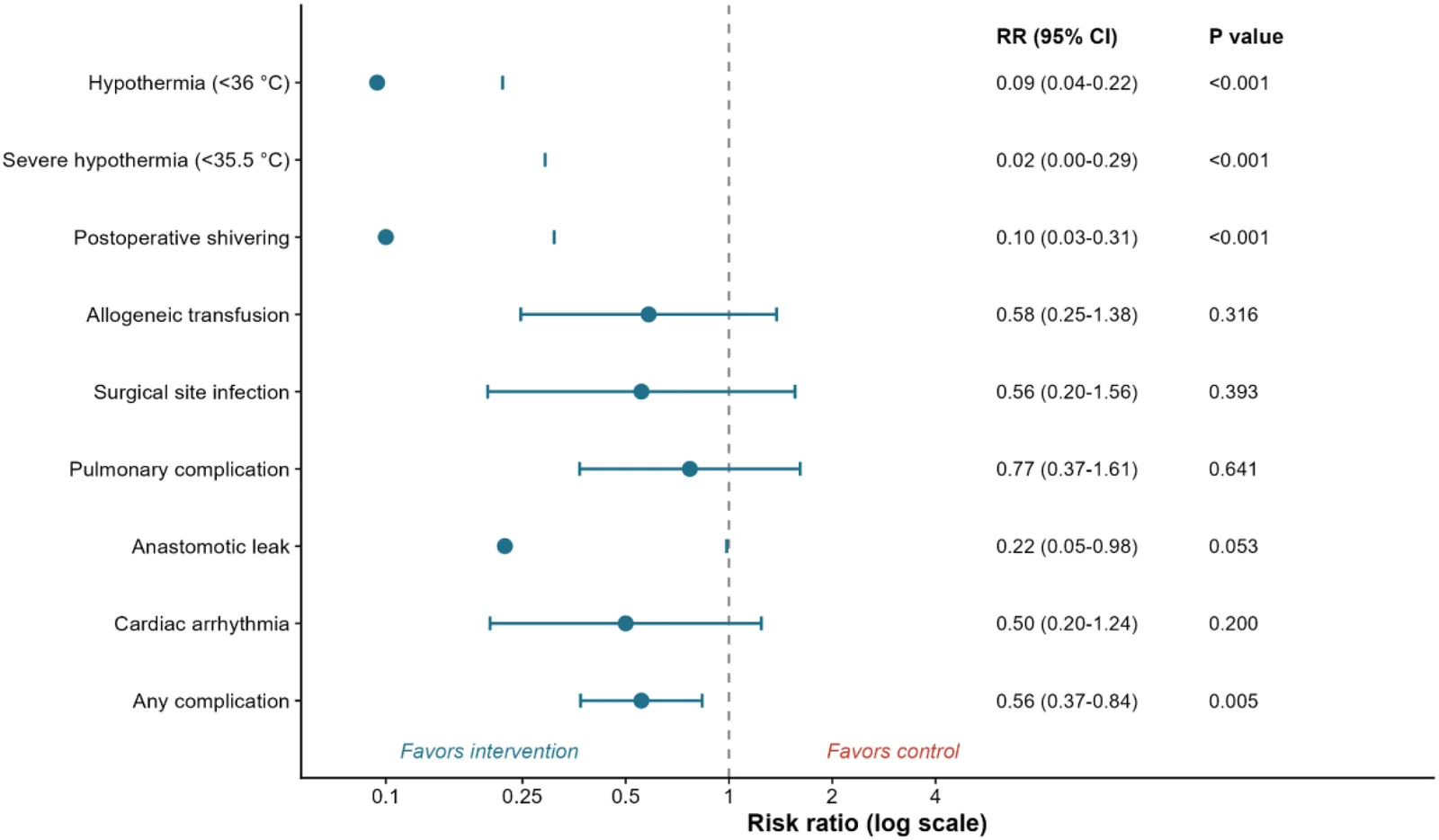
Forest plot of risk ratios for the primary endpoint and selected secondary outcomes. Values are shown on a logarithmic scale with 95% confidence intervals.

### Postoperative complications, adjusted analysis, and subgroup effects

Thirty-day postoperative complications are summarized in **Table 3** and **Figure 6**. The composite endpoint of any complication occurred in 62.1% of control patients and 34.5% of intervention patients (P = 0.005). Individual complications also favored the intervention group numerically. Anastomotic leak was less frequent in the intervention arm (3.4% vs 15.5%), although the study was not powered to detect differences for each individual component. Surgical site infection, pulmonary complications, and cardiac arrhythmia also occurred less often with protocolized warming, but these differences did not reach statistical significance individually. Resource use paralleled the clinical findings. ICU stay was shorter in the intervention group (1.9 ± 0.8 vs 2.4 ± 0.8 days; P < 0.001), total hospital stay was reduced by 1.3 days on average (P = 0.010), and in-hospital cost was lower by 8,911 CNY (P < 0.001). Across the full cohort, greater hypothermia burden was associated with longer hospital stay (Pearson r = 0.31, P < 0.001) (**Figure 7**). Because complications drive both hospitalization and expenditure, the shorter stay and lower cost in the intervention group are most plausibly mediated by the lower complication rate and should be interpreted as associated benefits rather than independent effects.

**Table 3:** Postoperative complications within 30 days.

Complication	Control (n = 58)	Intervention (n = 58)	P value
Surgical site infection, n (%)	9 (15.5%)	5 (8.6%)	0.393
Pulmonary complication, n (%)	13 (22.4%)	10 (17.2%)	0.641
Anastomotic leak, n (%)	9 (15.5%)	2 (3.4%)	0.053
Cardiac arrhythmia, n (%)	12 (20.7%)	6 (10.3%)	0.200
Any complication, n (%)	36 (62.1%)	20 (34.5%)	0.005

**Figure 6:**
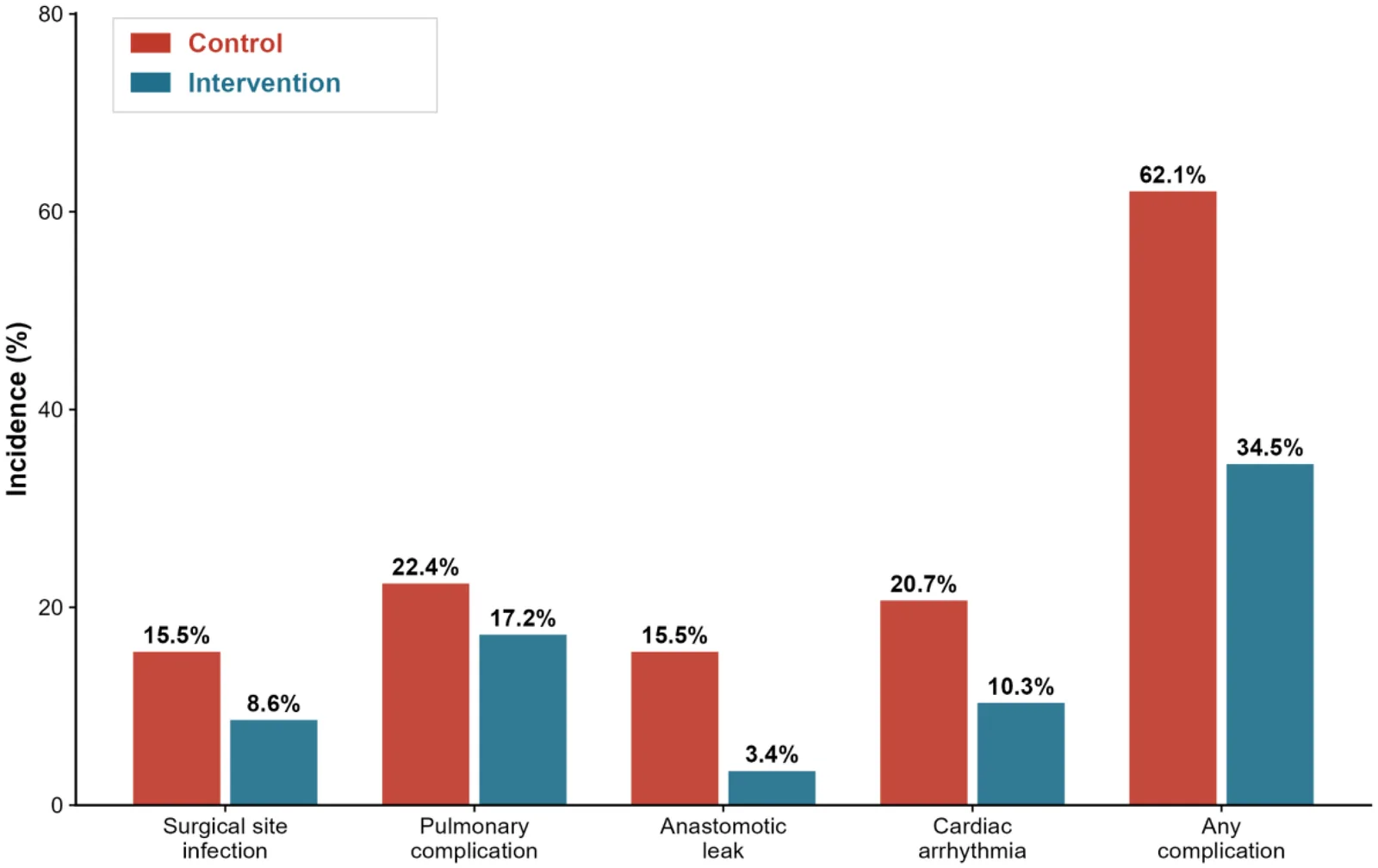
Incidence of postoperative complications in the control and intervention groups.

**Figure 7:**
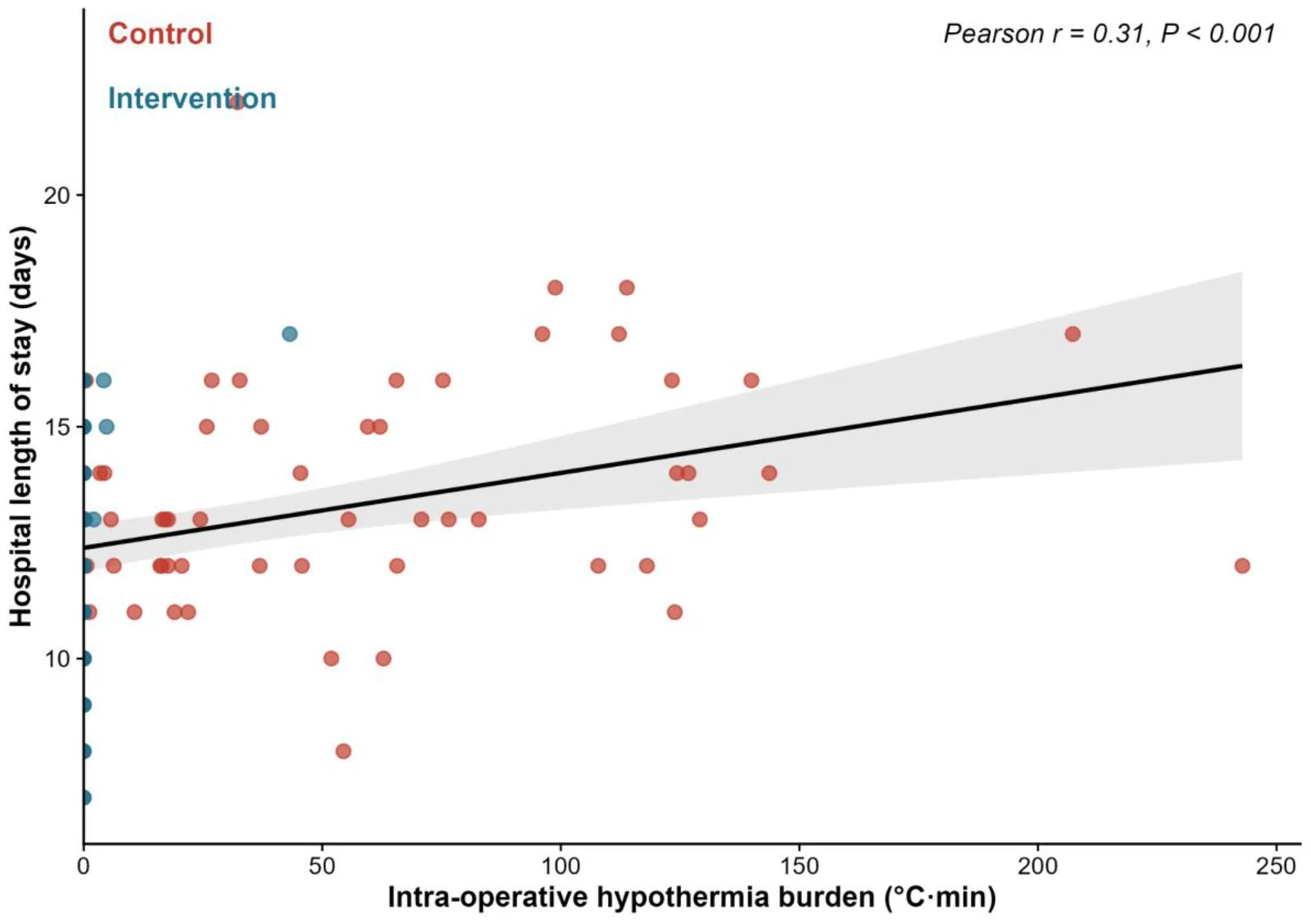
Association between hypothermia burden and postoperative hospital stay.

The adjusted analyses for the primary endpoint are shown in **Table 4**. Because 53 of 58 control patients and only 5 of 58 intervention patients reached the primary endpoint, the data are close to complete separation, and conventional maximum-likelihood logistic regression produced extreme, unstable estimates (adjusted OR < 0.001). The Firth penalized model, which is designed for sparse-data settings, yielded an adjusted OR of 0.004 (95% profile-penalized-likelihood CI 0.000-0.024; P < 0.001) for the intervention and an adjusted OR of 0.50 (95% CI 0.001-0.74; P < 0.001) per 0.1 °C increase in baseline core temperature; no other covariate was significantly associated with the outcome. Because odds ratios overstate effect size for a common outcome, we also estimated the adjusted risk ratio (0.10, 95% CI 0.04-0.23) and the adjusted risk difference (-81 percentage points, 95% CI -92 to -71), both consistent with the unadjusted comparison. A *post hoc* sensitivity model additionally adjusting for preoperative hemoglobin did not materially change the treatment estimate (adjusted OR 0.005, 95% CI 0.000-0.030). Prespecified subgroup analyses showed a consistent benefit across age, sex, BMI, ASA class, surgical approach, and operative duration (**Figure 8**). No subgroup suggested attenuation or reversal of the treatment effect.

**Table 4:** Adjusted analyses for intraoperative hypothermia (Firth penalized logistic regression).

Covariate	Adjusted OR	95% CI	P value
Intervention group (vs. control)	0.004	0.000 to 0.024	<0.001
Age (per year)	0.959	0.853 to 1.059	0.419
Female sex (vs. male)	0.712	0.108 to 4.112	0.706
BMI (per kg/m²)	0.979	0.733 to 1.300	0.880
ASA class III (vs. II)	1.719	0.409 to 7.815	0.457
Operative duration (per min)	1.006	0.989 to 1.022	0.501
Operating-room temperature (per °C)	0.817	0.234 to 2.485	0.729
Baseline core temperature (per 0.1 °C)	0.50	0.001 to 0.74	<0.001

Estimates are from Firth penalized logistic regression with profile-penalized-likelihood confidence intervals and likelihood-ratio P values, adjusting for the prespecified covariates listed. Conventional maximum-likelihood estimation was unstable because of near-complete separation of the outcome (53/58 vs 5/58). Complementary models gave an adjusted risk ratio of 0.10 (95% CI 0.04-0.23; modified Poisson regression with robust variance) and an adjusted risk difference of -81 percentage points (95% CI -92 to -71). The estimate for baseline core temperature is expressed per 0.1 °C increment. Separation, convergence, standard errors, and influential observations were examined before interpretation. A *post hoc* sensitivity model additionally including preoperative hemoglobin gave an adjusted OR of 0.005 (95% CI 0.000-0.030) for the intervention. OR, odds ratio; CI, confidence interval.

**Figure 8:**
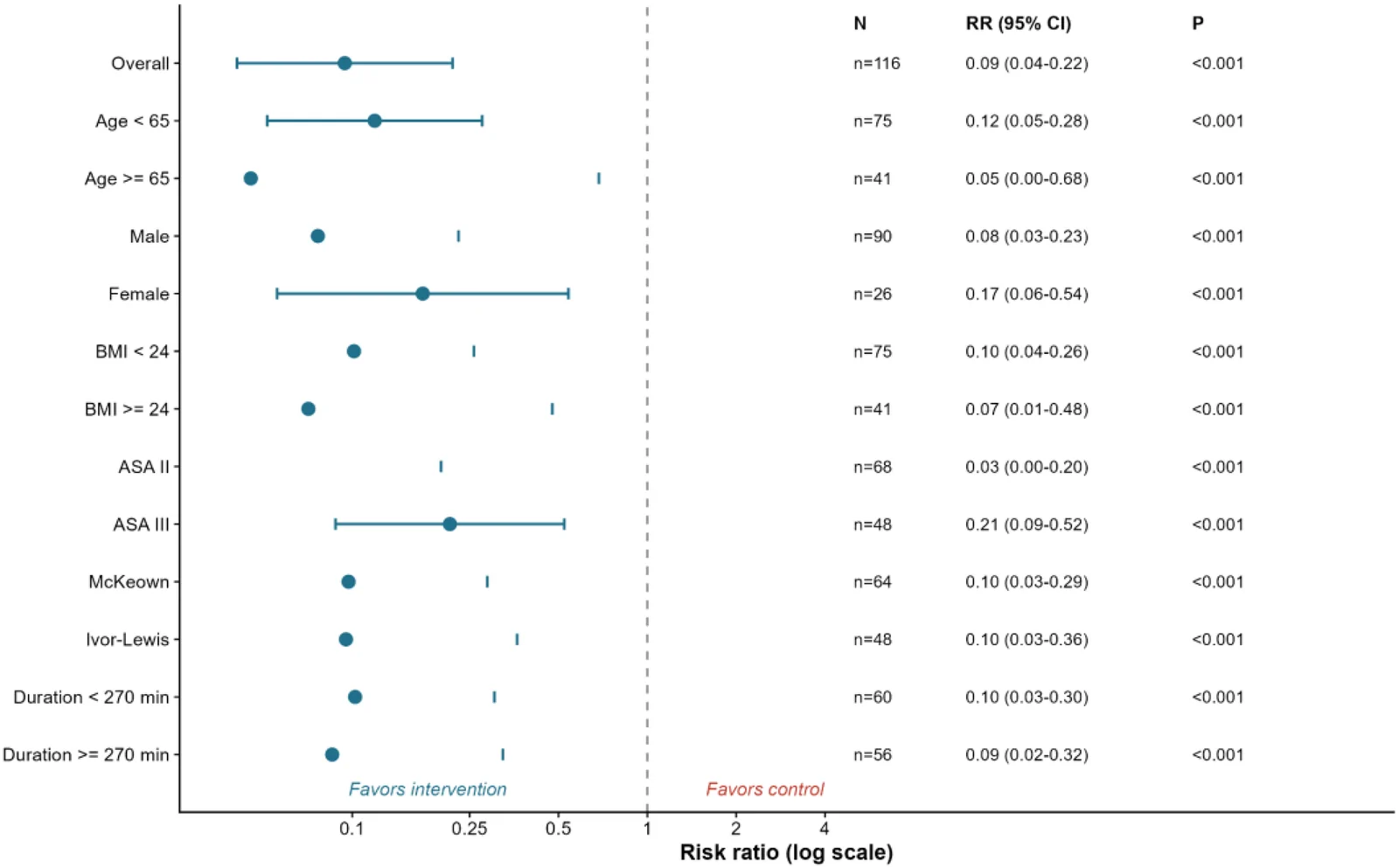
Predefined subgroup analysis for the primary endpoint. Risk ratios are shown with 95% confidence intervals.

## Discussion

In this retrospective comparative cohort study, a time-segmented, feedback-guided tiered warming protocol was associated with greatly reduced intraoperative hypothermia during radical esophagectomy. The effect was large rather than incremental: patients managed with the protocol rarely became hypothermic, none developed severe hypothermia, and time spent below 36 °C was nearly eliminated. These thermal differences were accompanied by less postoperative shivering, faster extubation, and shorter PACU recovery — outcomes with a direct physiological link to core temperature. The lower overall complication rate, shorter hospitalization, and lower cost observed in the protocol group should be interpreted more cautiously: these are associations from a retrospective, non-randomized comparison and remain susceptible to residual confounding, so causality for these downstream outcomes cannot be established by this study and requires confirmation in prospective multicenter studies.

The size of the treatment effect is consistent with the thermal demands of esophagectomy itself. The procedure combines anesthetic-induced redistribution, prolonged exposure of the thoracic and abdominal cavities, fluid administration, and long operative duration. Against that background, the high hypothermia rate in the control group is not unexpected and aligns with prior esophagectomy studies showing worse early outcomes among colder patients (15, 16). The present results are also in line with the broader perioperative literature linking hypothermia to wound infection, transfusion, delayed recovery, and longer hospitalization. The neutral cardiovascular finding in PROTECT should be interpreted in that context: it was derived from a heterogeneous non-cardiac population and does not address the narrower question of how best to prevent sustained heat loss during prolonged thoracoabdominal cancer surgery (20). An important design consideration is that the intervention was a multicomponent bundle: compared with the control strategy, it included prewarming, a higher baseline fluid-warming temperature, a guaranteed minimum ambient temperature, and earlier, protocol-driven escalation. The study therefore compared an intensified, protocolized warming bundle against conservative conventional care, and the independent contributions of prewarming, fluid temperature, ambient temperature, and the time-segmented tiered escalation cannot be distinguished in this design. The observed benefit should accordingly be attributed to the protocol as a whole rather than to the threshold scheme or any feedback mechanism in isolation; component-level or factorial studies would be needed to isolate the active ingredients.

The benefit of the present protocol appears to derive not only from warming intensity, but from earlier recognition and graded escalation. It should be emphasized that the protocol was not an automated closed-loop system: temperature acquisition, threshold assessment, and device adjustment were all performed manually by clinical staff following the written algorithm, and we have therefore described the strategy as feedback-guided and protocolized rather than closed-loop. Experimental data show that lower temperature impairs coagulation enzyme activity and platelet function, and clinical reviews have long linked perioperative cooling to postoperative shivering (25, 26). Editorial and narrative reviews have likewise argued that temperature management should be handled as an active perioperative process rather than a late rescue measure (27–29). In long thoracoabdominal surgery, waiting until the patient has already crossed 36 °C is a delayed trigger, particularly during the early redistribution phase described in classical thermoregulation work (6, 30). The broader perioperative literature on mild hypothermia prevention points in the same direction (31).

These findings also matter at the system level. In a country where esophageal cancer remains common and esophagectomy is performed at high volume, perioperative temperature control is also a quality-of-care issue. Compliance with established normothermia process measures has been associated with better clinical outcomes (32). Economic analyses suggest that structured normothermia protocols can reduce excess hospital stay and overall expenditure (33). Evidence from colectomy, open colon surgery, and preoperative warming trials supports the same general interpretation, linking better temperature control with lower wound-related morbidity or improved local thermal preservation (34–37). This study has several limitations. It was a single-center retrospective comparison in which the warming strategy was not randomly assigned, so residual confounding — including confounding by indication — cannot be excluded. The intervention was also a multicomponent bundle, so no single element can be credited with the effect. Although operations were performed by a small number of experienced teams working to standardized techniques and postoperative care followed a uniform institutional pathway, unmeasured differences in surgical difficulty may still have contributed to the difference in complications, and hospital stay and cost are largely downstream of complications; these associations are therefore hypothesis-generating. Finally, the near-complete separation of the primary endpoint required penalized-likelihood estimation, so the adjusted risk ratio and risk difference should be preferred to the Firth odds ratios; the sample was not powered for individual complications; and follow-up and costing were limited to 30 days and to direct in-hospital expenditure respectively. These data support viewing temperature management in major cancer surgery as a structured perioperative process rather than a background task. A low-cost protocol that reduces complications, shortens hospitalization, and improves consistency of care warrants consideration in high-volume cancer centers.

## Conclusion

A time-segmented, feedback-guided tiered warming protocol was associated with substantially better thermal control during radical esophagectomy and with better short-term recovery, fewer overall complications, shorter hospitalization, and lower cost; these associations warrant confirmation in prospective multicenter studies with detailed control of surgical and postoperative variables. Because the protocol relies on standard perioperative equipment, it may be a scalable perioperative quality-improvement measure for high-risk oncologic surgery.

## Data Availability

The original contributions presented in the study are included in the article/supplementary material. Further inquiries can be directed to the corresponding author.
